# Implication from thyroid function decreasing during chemotherapy in breast cancer patients: chemosensitization role of triiodothyronine

**DOI:** 10.1186/1471-2407-13-334

**Published:** 2013-07-06

**Authors:** Jianbo Huang, Liangbin Jin, Guangyan Ji, Lei Xing, Chaobo Xu, Xiong Xiong, Hongyuan Li, Kainan Wu, Guosheng Ren, Lingquan Kong

**Affiliations:** 1 Department of Endocrine & Breast Surgery, the First Affiliated Hospital of Chongqing Medical University, You Yi Rd. 1, Chongqing, People’s Republic of China

**Keywords:** Breast cancer, Thyroid hormones, Non-thyroidal illness syndrome, Chemotherapeutic efficacy

## Abstract

**Background:**

Thyroid hormones have been shown to regulate breast cancer cells growth, the absence or reduction of thyroid hormones in cells could provoke a proliferation arrest in G0-G1 or weak mitochondrial activity, which makes cells insensitive to therapies for cancers through transforming into low metabolism status. This biological phenomenon may help explain why treatment efficacy and prognosis vary among breast cancer patients having hypothyroid, hyperthyroid and normal function. Nevertheless, the abnormal thyroid function in breast cancer patients has been considered being mainly caused by thyroid diseases, few studied influence of chemotherapy on thyroid function and whether its alteration during chemotherapy can influence the respose to chemotherapy is still unclear. So, we aimed to find the alterations of thyroid function and non-thyroidal illness syndrome (NTIS) prevalence druing chemotherapy in breast cancer patients, and investigate the influence of thyroid hormones on chemotherapeutic efficacy.

**Methods:**

Thyroid hormones and NTIS prevalence at initial diagnosis and during chemotherapy were analyzed in 685 breast diseases patients (369 breast cancer, 316 breast benign lesions). The influence of thyroid hormones on chemotherapeutic efficacy was evaluated by chemosensitization test, to compare chemotherapeutic efficacy between breast cancer cells with chemotherapeutics plus triiodothyronine (T_3_) and chemotherapeutics only.

**Results:**

In breast cancer, NTIS prevalence at the initial diagnosis was higher and increased during chemotherapy, but declined before the next chemotherapeutic course. Thyroid hormones decreased signigicantly during chemotherapy. T_3_ can enhance the chemosensitivity of MCF-7 to 5-Fu and taxol, with progression from G0-G1 phase to S phase. The similar chemosensitization role of T_3_ were found in MDA-MB-231. We compared chemotherapeutic efficacy among groups with different usage modes of T_3_, finding pretreatment with lower dose of T_3_, using higher dose of T_3_ together with 5-Fu or during chemotherapy with 5-Fu were all available to achieve chemosensitization, but pretreatment with lower dose of T_3_ until the end of chemotherapy may be a safer and more efficient therapy.

**Conclusions:**

Taken together, thyroid hormones decreasing during chemotherapy was found in lots of breast cancer patients. On the other hand, thyroid hormones can enhance the chemotherapeutic efficacy through gatherring tumor cells in actively proliferating stage, which may provide a new adjuvant therapy for breast cancer in furture, especially for those have hypothyroidism during chemotherapy.

## Background

Thyroid hormones, which function in cell development, differentiation, growth, and other aspects of metabolism, are regulated by hypothalamic-pituitary axis. In cancer patients, thyroid function is thought to be vulnerable to chemotherapy, as hypothalamic-pituitary axis is active and chemotherapy is systemic therapy for patients. The influence of chemotherapy on thyroid function was just seen as a late effect [1,2], mainly presenting hypothyroidism. Nevertheless, thyroid function decreasing does not occur in all cancer patients receiving chemotherapy, thyroxine (T_4_), T_3_ and reverse triiodothyronine (rT_3_) levels of patients with non-seminoma testicular carcinoma increased significantly during chemotherapy [3], some choriocarcinoma patients even suffered thyroid crisis during chemotherapy [4,5], the cause may be the high levels of human chorionic gonadotrophin (HCG), producing biochemical and clinical hyperthyroidism [6]. While, non-seminoma testicular carcinoma and choriocarcinoma are rare human malignancies that are highly curable with chemotherapy or combination of surgery and chemotherapy, even with widespread metastases. For breast cancer patients, studies did not directly show chemotherapy can influence thyroid function, only found some results indicative hypothyroidism, such as elevated concentrations of thyroid stimulating hormone (TSH), thyroperoxidase antibody (anti-TPO), thyroglobulin antibody (anti-Tg) [7] and decreased T_3_ uptake levels [8]. Few studied the alteration of thyroid function in breast cancer patients during chemotherapy, the time that chemotherapeutic reagents take effect in.

On the other hand, chemotherapeutic efficacy can be influenced by certain endocrine hormones or related therapy. In vivo, rat treated with growth hormone and chemotherapy had better efficacy than those treated with chemotherapy only [9]. In clinic, tamoxifen, which competitively combine with estrogen receptor, can not be concurrently used with chemotherapy for breast cancer patients, as the cancer growth inhibition by tamoxifen makes tumor cells insensitive to chemotherapy. Interestingly, choriocarcinoma and testicular carcinoma are highly curable with chemotherapy, even with widespread metastases, they have elevated thyroid hormones in common during chemotherapy, is better chemotherapeutic efficacy related with elevated thyroid hormones? Whether T_3_ can enhance sensitivity of breast cancer cells to chemotherapy is unclear.

In this study, 1698 serum samples in 685 cases of breast diseases, including 369 cases of breast cancer and 316 cases of breast benign lesions were analyzed to study the thyroid hormones and NTIS prevalence in breast cancer patients at initial diagnosis and during chemotherapy. According to the changes of thyroid hormones during chemotherapy, the effect of T_3_ on sensitity of breast cancer cells to chemotherapy was studied.

## Methods

### Patients and samples

A total of 685 female patients with breast diseases admitted in the First Affiliated Hospital of Chongqing Medical University, with median age of 49 years (range 17–81 years) was studied, including 316 cases of breast benign lesions, such as fibroadenoma, mastopathy, intraductal papilloma, lipoma, etc. and 369 cases of breast carcinoma, among which 224 cases were initially diagnosed with breast carcinoma. All breast diseases were confirmed by the pathology. No history of thyroidal diseases or liver, renal dysfunction was found in these patients. All breast cancer patients were treated with standard chemotherapy. A total of 1698 samples were collected from all patients on the first admission and / or 1 to 3 days before and / or 1 to 3 days after each cycle chemotherapy.

Ethical approval for this study and agreement by all patients were obtained from the Biomedicine Ethics Committee of The First Affiliated Hospital of Chongqing Medical University. Each subject signed an agreement of participation in this study that was approved by Biomedicine Ethics Committee of The First Affiliated Hospital of Chongqing Medical University and consented to publish. The protocol of the study adhered to the tenets of the Declaration of Helsinki and was approved by the local ethics committee.

### Thyroid function assay

T_3_, T_4_, free triiodothyronine (FT_3_), free thyroxine (FT_4_), TSH were detected by UnicelTM DXI 800 with chemiluminescence methods in the Laboratory of Endocrinology of the First Affiliated Hospital of Chongqing Medical University.

### Cell line and cell culture

The MCF-7, MDA-MB-231 breast cancer cell lines were supported by Molecular Oncology and Epigenetics Laboratory, the First Affiliated Hospital of Chongqing Medical University. The cell lines were maintained in RPMI 1640 (Gibco-BRL, Karlsruhe, Germany) supplemented with 10% fetal bovine serum (FBS) (PAA Laboratories, Linz, Austria), 100 U/ml penicillin and 100 mg/ml streptomycin, at 37°C in a humidified atmosphere containing 5% CO_2_.

### Chemosensitization test and reagents

**Figure 1:**
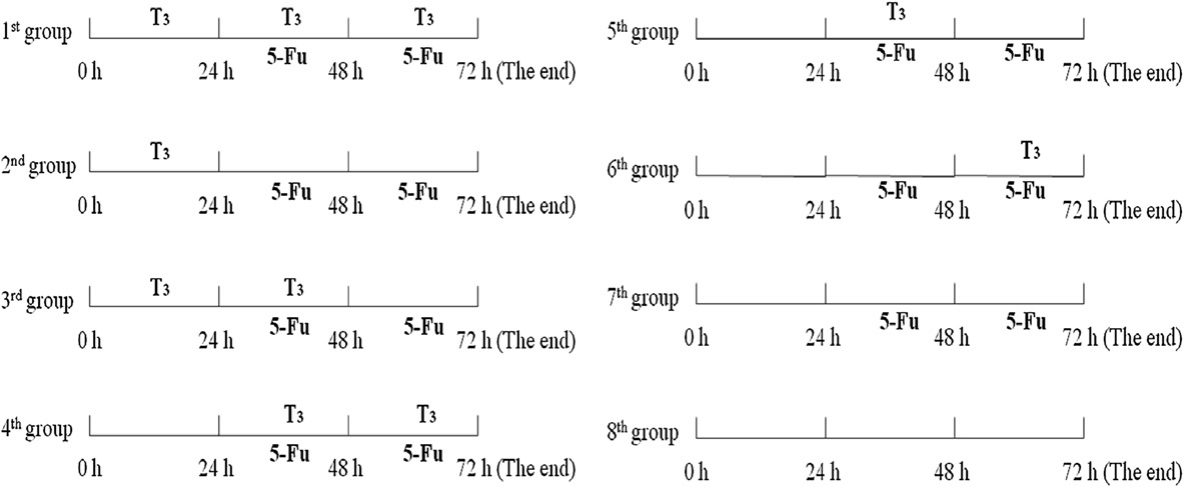
**The illustration for chemosensitization test with different group.** The 1st to 3rd groups were pretreated with T_3_ for 24 h, then treated with 5-Fu for 48 h, the depriving times of T_3_ were different, the 1st group was kept with T_3_ until test end, T_3_ of the 2nd group was deprived at the start of 5-Fu, and T_3_ of the 3rd group was deprived after using 5-Fu for 24 h. After 24 h without T_3_ pretreatment, the 4th to 6th groups were also treated with 5-Fu for 48 h. The 4th group was added with T_3_ at the start of 5-Fu until test end, T_3_ of the 5th group was added at the start of 5-Fu and deprived after using for 24 h, T_3_ of the 6th was added when 5-Fu was added for 24 h. After 24 h without T_3_ pretreatment, the 7th group was treated with 5-Fu only for 48 h, and without treating with T_3_. The 8th group was set as control group, without treating with T_3_ or 5-Fu.

The chemosensitization role of T_3_ (Sigma Aldrich, Italy) is reflected by changes of sensitivity of breast cancer cells to 5-fluorouracil (5-Fu) (Xu Dong, China) or Taxol (De Bao, China). The concentration of T_3_ used for chemosensitization test ranged from 2 ng/ml to 32 ng/ml, 5-Fu and Taxol were used at the IC_50_ concentration for each cell line (5-Fu: 13 μmol/L for MCF-7, 8 μmol/L for MDA-MB-231; Taxol: 0.4 μmol/L for MCF-7, 0.5 μmol/L for MDA-MB-231). According to different concentrations of T_3_, the chemosensitization test comprised nine independent tests, each contained eight groups with a specific T_3_ concentration (Figure 1). Before chemosensitization test began, the MCF-7 cells in logarithmic growth phase were trypsinized and seeded at a density of 3000/well into 96-well plates at 37°C in 5% incubator. After 21 h, the chemosensitization test began.

The 1st to 3rd groups were pretreated with T_3_ for 24 h, then treated with 5-Fu for 48 h, the depriving times of T_3_ were different, the 1st group was kept with T_3_ until test end, T_3_ of the 2nd group was deprived at the start of 5-Fu, and T_3_ of the 3rd group was deprived after using 5-Fu for 24 h.

After 24 h without T_3_ pretreatment, the 4th to 6th groups were also treated with 5-Fu for 48 h. The 4th group was added with T_3_ at the start of 5-Fu until test end, T_3_ of the 5th group was added at the start of 5-Fu and deprived after using for 24 h, T_3_ of the 6th group was added when 5-Fu was added for 24 h.

After 24 h without T_3_ pretreatment , the 7th group was treated with 5-Fu only for 48 h, and without treating with T_3_. The 8th group was set as control group, without treating with T_3_ or 5-Fu.

### Cell proliferation assay and inhibition ratio calculation

Before cell proliferation assay, wells of all groups were washed with PBS for two times, replaced with fresh culture medium, and set one new group with culture medium only as blank control. Cell counting kit-8 (CCK8) (Dojindo Laboratories, Japan) was used to evaluate the viability of cells in all groups according to the instructions of the manufacturer. The final concentration used for assay was 10%, after incubation with cells for 1 h at 37°C to allow CCK8 to form formazan crystals by reacting with metabolical active cells, the OD value was measured in a microplate reader at 450 nm. The inhibition ratio was calculated by following formula: 1-OD value of treated group / OD value of control group. All experiments were repeated at least three times by same person.

### Flow cytometry analysis of cell cycle

Flow cytometry analysis of cell cycle was implemented as previously reported [10]. For cell cycle analysis, cells were fixed in ice-cold 70% ethanol and stained with propidium iodide (PI). The cell cycle profiles were assayed using the Elite ESP flow cytometry at 488 nm, and the data were analyzed using the CELL Quest software (BD Biosciences, San Jose, CA). Flow cytometric analysis was immediately performed.

### Statistical analysis

All statistical analyses were performed using SPSS 18.0 statistical software. The Student’s t-test was used to compare thyroid function between patients of benign lesions and cancer, the comparison of thyroid function between postchemotherapy and prechemotherapy, thyroid function between two consecutive prechemotherapies were performed with paired statistical comparison, the nonparametric test was used when equal variances is not assumed. The Anova or the nonparametric tests were applied to compare the proportion of tumor cells in S phase between treated and control group, inhibition ratios among seven different treated groups. Results of thyroid functions were presented as mean ± SD, values of *P*≤0.05 were considered significant.

## Results

### Thyroid function of patients with breast benign lesions and breast cancer

A total of 224 serum samples of breast cancer patients and 316 samples of breast benign lesion patients at initial diagnosis were analyzed for thyroid hormones. Thirty-seven of 224 breast cancer patients were diagnosed as NTIS (either FT_3_ or FT_4_ is below the normal), of which the morbidity was higher than that in breast benign lesions patients (16.5% vs 7.3%). The mean concentrations of T_3_, FT_3_ in breast cancer patients were lower than that in breast benign lesions patients, however, only difference of T_3_ between them was statistically significant (*P*<0.05). On contrary, the mean concentrations of T_4_ and TSH were higher in breast cancer patients, but only difference of T_4_ between them was statistically significant (*P*≤0.05) (Table 1).

**Table 1:** Comparison of thyroid function between patients of breast benign lesions and cancer on initial diagnosis (mean ± SD)

**Thyroid functions**	**Breast benign lesions patients (n=316)**	**Breast cancer patients (n=224)**	***p *value**
T_3_ (ng/ml)	1.22±0.23^*^	1.16±0.25	0.002
T_4_ (μg/dl)	7.12±1.22^*^	7.34±1.36	0.050
FT_3_ (pg/ml)	2.92±0.43	2.87±0.38	0.149
FT_4_ (ng/dl)	0.89±0.13	0.89±0.18	0.650
TSH (μIU/ml)	3.16±4.92	3.92±8.40	0.187
**Clinicopathological features of breast cancer**		Number	
Tumor size	T0 (impalpable)	11	
	T1 (≤2 cm)	84	
	T2 (>2 cm, ≤5 cm)	105	
	T3 (>5 cm)	19	
	Unknown	5	
Tumor grade	I	11	
	II	188	
	III	19	
	Unknown	6	
Clinical stage	0	9	
	I	63	
	II	132	
	III	11	
	IV	4	
	Unknown	5	

### Alterations of thyroid function in breast cancer patients during chemotherapy

Blood samples from 180 cases of breast cancer patients were analyzed in this part. Eighty-two point two percent (82.2%) patients suffered NTIS during chemotherapy. T_3_, T_4_, FT_3_, TSH decreased and FT_4_ increased significantly during chemotherapy compared with prechemotherapy (*P*<0.05) (Table 2).

**Table 2:** Comparison of thyroid function in breast cancer patients between prechemotherapy and postchemotherapy (mean ± SD)

**Thyroid function**	**Prechemotherapy (n=180)**	**Postchemotherapy (n=180)**	***p *value**
T_3_ (ng/ml)	1.16±0.27	0.80±0.27	<0.001
T_4_ (μg/dl)	7.30±1.23	6.87±1.57	<0.001
FT_3_ (pg/ml)	2.89±0.57	2.10±0.49	<0.001
FT_4_ (ng/dl)	0.85±0.14	0.90±0.19	0.019
TSH (μIU/ml)	2.78±2.16	1.00±1.31	<0.001
Age (y)	49.8		
**Clinicopathological features**		Number	
Tumor size	T0 (impalpable)	7	
	T1 (≤2 cm)	66	
	T2 (>2 cm, ≤5 cm)	90	
	T3 (>5 cm)	12	
	Unknown	5	
Tumor grade	I	8	
	II	150	
	III	13	
	Unknown	9	
Clinical stage	0	9	
	I	46	
	II	107	
	III	7	
	IV	5	
	Unknown	6	
Chemotherapy regimens	TEC	158	
	CEF	13	
	NP	8	
	Unknown	1	

TEC: Docetaxel, Epirubicin, Cyclophosphamide.

CEF: Cyclophosphamide, Epirubicin, Fluorouracil.

NP: Navelbine, Cisplati.

### Comparison of thyroid function between two consecutive prechemotherapies

A total of 456 samples were analyzed between two consecutive prechemotherapies, no significant differences of thyroid function were found (*P*>0.05) (Table 3).

**Table 3:** Comparison of thyroid function in breast cancer patients between two consecutive prechemotherapies (mean ± SD)

**Thyroid function**	**Previous prechemotherapy (n=228)**	**Next prechemotherapy (n=228)**	***p *value**
T_3_ (ng/ml)	1.14±0.23	1.18±0.25	0.083
T_4_ (μg/dl)	7.11±1.20	7.07±1.19	0.553
FT_3_ (pg/ml)	2.84±0.37	2.90±0.67	0.188
FT_4_ (ng/dl)	0.85±0.14	1.27±6.66	0.736
TSH (μIU/ml)	3.11±3.35	2.76±2.30	0.064
Age (y)	49.5		
**Clinicopathological features**		Number	
Tumor size	T0 (impalpable)	7	
	T1 (≤2 cm)	90	
	T2 (>2 cm, ≤5 cm)	113	
	T3 (>5 cm)	16	
	Unknown	2	
Tumor grade	I	14	
	II	192	
	III	13	
	Unknown	9	
Clinical stage	0	6	
	I	72	
	II	130	
	III	12	
	IV	6	
	Unknown	2	
Chemotherapy regimens	TEC	202	
	CEF	12	
	NP	10	
	Unknown	4	

TEC: Docetaxel, Epirubicin, Cyclophosphamide.

CEF: Cyclophosphamide, Epirubicin, Fluorouracil.

NP: Navelbine, Cisplatin.

### The chemosensitization test of triiodothyronine

**Figure 2:**
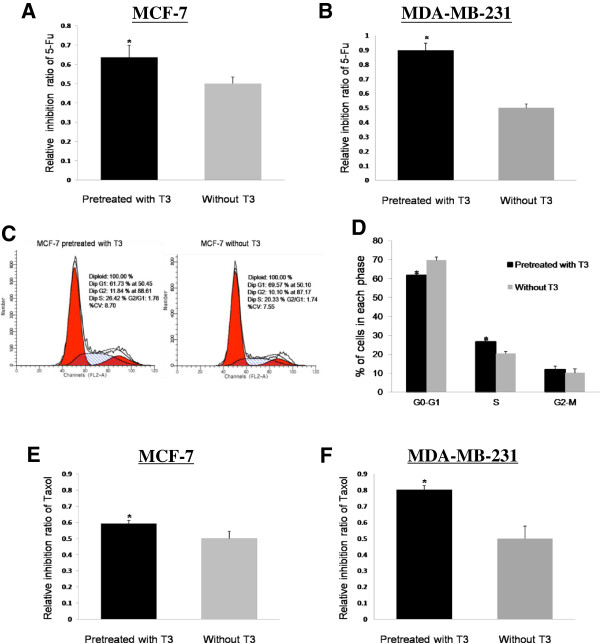
**Pretreating MCF-7 or MDA-MB-231 with T**_**3 **_**enhanced the sensitivity of breast cancer cells to 5-Fu and Taxol. (A)** After using 4 ng/ml T_3_ to pretreat MCF-7 for 24 h, the inhibition ratio was higher in MCF-7 pretreated with T_3_, compared with MCF-7 with 5-Fu only, without T_3_ pretreatment, * *P*<0.05. **(B)** The inhibition ratio was higher in MDA-MB-231 pretreated with T_3_, compared with those with 5-Fu only, without T_3_ pretreatment, * *P*<0.05. **(C**&**D)** MCF-7 pretreated with T_3_ for 24 h showed significantly increased proportion of cells in S phase, and significantly decreased proportion of cells in G0-G1 phase, both compared with those of MCF-7 without T_3_ pretreatment, * *P*<0.05. **(E**&**F)** After using 24 ng/ml T_3_ to pretreat MCF-7 or MDA-MB-231 for 24 h, the inhibition ratio was higher in cancer cells pretreated with T_3_, compared with those with Taxol only, without T_3_ pretreatment, * *P*<0.05.

The thyroid functions decreasing during chemotherapy indicated adding thyroid hormones may enhance the sensitivity of tumor cells to chemotherapy, as T_3_ can induce cells progression from G0-G1 phase to S phase [11] or enhance mitochondrial activity [12] which makes tumor cells more sensitive to chemotherapy. So, as the specific chemotherapeutic reagent for killing tumor cells in S phase, 5-Fu was choosed to study the change of sensitivity of breast cancer cells when T_3_ was present. Three groups were set up, cells were seeded in quintuplicate in 96-well plate at a density of 3×10^3^ cells/well. The 1st group was treated with T_3_ and 5-Fu, the 2nd group was treated with 5-Fu only, the 3rd group was set as control group. We firstly used 4 ng/ml T_3_ to pretreat MCF-7 or MDA-MB-231 for 24 h, then 5-Fu was added and T_3_ were deprived, values of OD after 48 h were measured, the results showed that both MCF-7 and MDA-MB-231 pretreated with T_3_ were more sensitive to 5-Fu than those with 5-Fu only, without T_3_ pretreatment (*P*<0.05) (Figure 2A and 2B).

The 5-Fu especially targets tumor cells in S phase, to study whether enhancement of sensitivity of tumor cells to 5-Fu was related with increasing proportion of breast cancer cells in S phase, we further investigated the effect of T_3_ on MCF-7 cell cycle. Representative results of cell-cycle distribution in MCF-7 pretreated with T_3_ or without T_3_ were shown in Figure 2C. Flow cytometry analysis revealed a statistically significant increase in the number of T_3_ pretreated MCF-7 with S phase (*P*<0.05), accompanied with the increase in G2-M phase and decrease in G0-G1 phase (*P*<0.05) (Figure 2D). So, T_3_ enhanced the sensitivity of MCF-7 to 5-Fu, through promoting the cell cycle progression of MCF-7 from G0-G1 phase to S phase.

Besides discussing the effect of thyroid hormone on 5-Fu, we also observed whether T_3_ has the chemosensitization role for Taxol. Taxol works differently by arresting the cells in G2-M phase, considering a higher concentration of T_3_ may be more efficient in promoting breast cancer cells proliferation, we used 24 ng/ml T_3_ to pretreat MCF-7 or MDA-MB-231, the results showed that both MCF-7 and MDA-MB-231 pretreated with T_3_ were more sensitive to Taxol than those with Taxol only, without T_3_ pretreatment (*P*<0.05) (Figure 2E and 2F).

### The different usage modes of triiodothyronine on chemosensitivity of MCF-7

Considering the thyroid function decrease during chemotherapy, which may induce breast cancer cells retardant in G0 phase, and T_3_ could enhance chemosensitivity of MCF-7 through increasing proportion of cells in S phase, we thought T_3_ may be used as a new adjuvant therapy for breast cancer, and started to study the efficacy of different T_3_ using modes on chemosensitivity of MCF-7.

**Figure 3:**
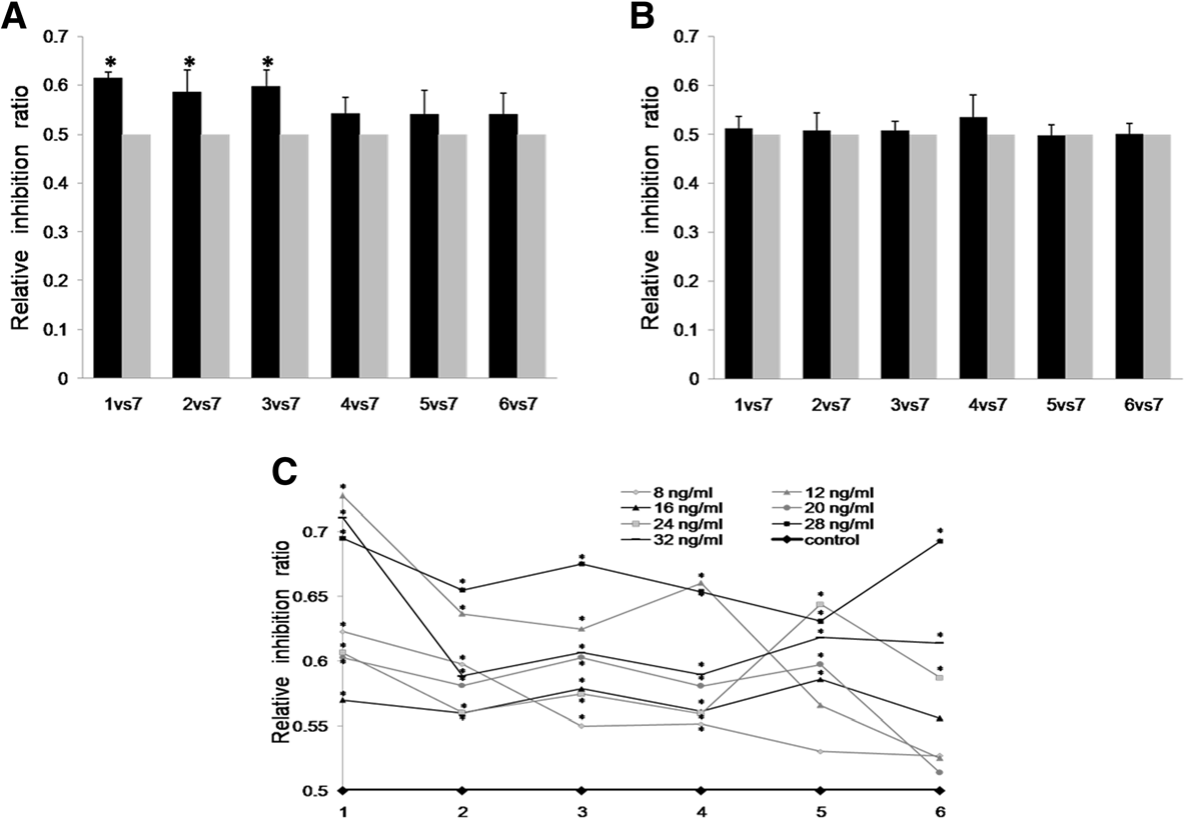
**The effect of different usage modes of T**_**3 **_**on sensitivity of MCF-7 to 5-Fu. (A)** Only sensitivity of MCF-7 to 5-Fu in the 1st to 3rd groups pretreated with 4 ng/ml T_3_ significantly increased, compared with that in group treated with 5-Fu only (7th group), * *P*<0.05; No significant difference of inhibition ratio was found between each of the 4th to 6th groups and 7th group, * *P*>0.05. **(B)** Pretreament MCF-7 with 2 ng/ml T_3_ did not show significant difference of inhibition ratio between each of 1st to 6th groups and 7th group, * *P*>0.05. **(C)** The inhibition ratio of different usage modes of T_3_, which ranged from 8 ng/ml to 32 ng/ml. The sensitivity of MCF-7 to 5-Fu in the 1st to 3rd groups pretreated with T_3_ all significantly increased, * *P*<0.05; The MCF-7 in 4th to 6th groups successively showed significantly increased chemosensitivity, compared with that of 7th group, * *P*<0.05, as the T_3_ dose elevating from 8 ng/ml to 32 ng/ml.

Eight groups were set as described in methods. As 4 ng/ml T_3_ had been proved to have chemosensitization role by us, we then firstly use 4 ng/ml with different usage modes to study the efficacy of T_3_ on chemosensitivity of MCF-7. Surprisingly, only sensitivity of MCF-7 to 5-Fu in the first three groups pretreated with T_3_ significantly increased (*P*<0.05), no chemosensitization was found in the 4th to 6th groups (Figure 3A).

Whether elevating the T_3_ dose can enhance the chemosensitivity of MCF-7 in 4th to 6th groups, those without T_3_ pretreatment, and decreasing T_3_ could reduce the chemosensitivity in the 1st to 3rd groups, those were pretreated with T_3_, were still unclear. So, we performed eight other chemosensitization tests with T_3_ at 2 ng/ml, 8 ng/ml, 12 ng/ml, 16 ng/ml, 20 ng/ml, 24 ng/ml, 28 ng/ml, 32 ng/ml, using the concentrations of T_3_ more than 4 ng/ml (8 ng/ml to 32 ng/ml) is to imitate the hyperthyroidism in choriocarcinoma patients receiving chemotherapy. The results showed 2 ng/ml T_3_ was not efficient enough to enhance the chemosensitivity of MCF-7 to 5-Fu in any group (*P*>0.05) (Figure 3B), which indicated the suitable dose of T_3_ is essential point for chemosensitization. On the contrary, the MCF-7 in 4th to 6th groups successively showed significantly increased chemosensitivity, compared with that of 7th group (*P*<0.05), as the T_3_ dose elevating from 8 ng/ml to 32 ng/ml (Figure 3C).

## Discussion

### Abnormal thyroid function in breast cancer patients at initial diagnosis

Many non-thyroidal illness may affect the thyoid function and induce NTIS [13], which is also called euthyroid sick syndrome (ESS) [14], low T_3_ or T_4_ syndrome [15], marked by reductions in both thyroid function and peripheral conversion of T_4_ to T_3_, presumed to reflect a homeostatic mechanism to conserve energy. TSH levels tend to be normal or slightly decreased in these patients, but the underlying mechanism of reduced thyroid function is unknown. In this study, the NTIS prevalence in breast cancer patients at initial diagnosis was higher than that in breast benign lesions patients (16.5% vs 7.3%). The high NTIS prevalence in breast cancer patients at initial diagnosis suggests that NTIS may be related with malignancy. Furthermore, it was found in this study that the mean concentration of FT_3_ in breast cancer patients at initial diagnosis was significantly lower than that in breast benign lesions patients, suggesting that thyroid hypofunction may occur in breast cancer patients. Galton VA [16] studied the effect of malignant tumor on the thyroid function in rats, implanted with Walker 256 carcinosarcoma cell line, results showed a reduction in the concentration of T_4_ in tumorous rats serum, compared with corresponding control rats, due to the increased deiodination and urinary excretion.

The causes for hypothyroidism in cancer patient may not only be associated with tumor necrosis factor, interleukin-1, interferon-γ [17,18], which inhibits the function of hypothalamus-pituitary-thyroid axis and peripheral conversion of T_4_ to T_3_, but also be associated with the self-protection mechanism, reducing the tissue metabolism to suppress the tumor growth.

### Alteration of thyroid function of breast cancer patients during chemotherapy

Chemotherapy is one of the most effective systemic therapies for cancers. In cancer patients, thyroid function is thought to be vulnerable to chemotherapy, as hypothalamic-pituitary axis is active and chemotherapy is systemic therapy for patients. The influence of chemotherapy on thyroid function was just seen as a late effect, mainly presenting hypothyroidism. Only few studied the alterations of thyroid function during chemotherapy, which induced abnormalities in thyroid hormones metabolism with a significant decrease in serum T_3_ concentration (NTIS) instead of overt thyroid disease in malignant hematologic disease [19]. Thyroid hypofunction was thought to be associated with inhibition of the liver thyroglobulin secretion [20] and hypothalamus-pituitary-thyroid axis function [7] by chemotherapy.

In this study, NTIS prevalence in breast cancer patients obviously increased during chemotherapy, compared with that at initial diagnosis (87.1% vs 16.5%) and then decreased to 15.4% before the next chemotherapy (prechemotherapy). The NTIS prevalence reduction from 16.5% to 15.4% indicated that chemotherapy alleviated the NTIS prevalence, as thyroid hypofunction is considered as risk for breast cancer occurrence [21] and poor prognosis [22], this also suggests that NTIS alleviation may be a good efficacy by chemotherapy. With significantly increased NTIS prevalence during chemotherapy, whether controlling it is better for enhancing chemotherapeutic efficacy is unclear.

Furthermore, during chemotherapy for breast cancer patients in this study, significant decreases of T_3_, T_4_, FT_3_ and TSH were found, compared with prechemotherapy (*P*<0.05). After chemotherapy, thyroid function began to recover, and there was no significant difference between two consecutive prechemotherapies (*P*>0.05), which indicated obvious thyroid function decreasing caused by chemotherapy mainly occurred during chemotherapy.

While there is still no detailed study on whether decrease of thyroid function goes against breast cancer chemotherapy or not. Decrease of thyroid function does not occur in all patients with malignant tumors during chemotherapy. In a prospective study, effects of chemotherapy on thyroid function in patients with non-seminoma testicular carcinoma were evaluted: Serum HCG was present in sixteen patients and absent in fifteen. HCG levels ranged from 10 to 30,000 μg/l, with a median value of 520 μg/l. And during chemotherapy, T_4_, T_3_ and rT_3_ levels increased significantly, but basal TSH levels and the TSH response to thyrotropin releasing hormone (TRH) decreased. Furthermore, after chemotherapy the increased T_4_ and lowered TSH levels returned to normal, while the FT_3_ level did not change either during or after chemotherapy [3], which suggested an unaltered hypothalamic / pituitary axis. Non-seminomatous germ-cell tumors (NSGCT) can comprise several different histological components, among which, choriocarcinoma, produces HCG. In NSGCT patients with high-serum HCG levels, hyperthyoidism has been recognized and is considered to be a paraneoplastic phenomenon. Hyperthyroidism frequently accompanies NSGCT with highly elevated HCG [23]. Recent investigations have clarified the structural homology not only in the HCG and TSH molecules but also in their receptors, and this homology suggests the basis for the reactivity of HCG with the TSH receptor [24,25]. Hyperthyroidism or increased thyroid function has been reported in many patients with trophoblastic tumors [26], either hydatidiform mole [27,28] or choriocarcinoma [25], for which the principal therapy is chemotherapy. With effective chemotherapy, their long term survival exceeds 95% [24,29]. Furthermore, hyperthyroidism can be cured after the healing of the trophoblastic tumors [30]. Meister LH et al. reported a 26-year-old pregnant woman suffered from choriocarcinoma with metastases to both lungs. HCG were more than 2.5×10^6^ mU/ml. Consistent with HCG-induced hyperthyroidism, the patient suffered from thyroid crisis with fever of 38.6°C, worsening respiratory distress, tachycardia of 140 bpm and mental confusion during the first cycle of chemotherapy. Then the patient was treated with antithyroid crisis, symptomatic and supportive treatments. After the 10th cycle of chemotherapy the patient was in good condition and free of metastatic lesions in her chest X-ray [5]. It was reported that thyroid hormones could significantly promote the tumor growth and metastases [31]. The effective chemotherapy of curable tumors (such as malignant hydatidiform mole, choriocarcinoma, etc.) may be associated with hyperthyroidism during chemotherapy, which may enhance the chemotherapeutic sensitivity by inducing tumor cells progression from G0-G1 phase into S phase or elevating the mitochondrial activity. While, in this study, thyroid function decreased significantly and majority of the breast cancer patients suffered NTIS during chemotherapy, which may be associated with decreased chemotherapy sensitivity. Therefore, based on the above analysis, it was hypothesized that increasing the thyroid function by giving thyroid hormones before and / or during chemotherapy to imitate the hyperthyroidism or high thyroid function state of some choriocarcinoma patients during chemotherapy may enhance the chemotherapeutic efficacy in breast cancer and other malignant tumor patients who suffer obviously decreased thyroid function or NTIS during chemotherapy.

### Effect of triiodothyronine on chemosensitization

The effect of thyroid hormones on chemotherapeutic efficacy has been rarely researched before, and whether adding thyroid hormones during chemotherapy is suitable for breast cancer patients is still unknown. Some studies found hypothyroidism may be the protective factor for cancers, as hypothyroidism can suppress tumor growth [32], in addition, Hercbergs et al. found that hypothyroidism could reduce the insulin-like growth factor 1, the antagonist of tamoxifen-induced cytotoxicity, to prolong the survival in recurrent high grade glioma patients [33]. This above mentioned perspective is different from our hypotheses, since we think hyperthyroid function is beneficial for treatment efficacy when patients receive chemotherapy. If patients of cancers reveive no chemotherapy, the status of hypothyroidism will be better for prognosis. Choriocarcinoma is curable with chemotherapy only, long term survival of these patients exceeds 95%, the underlying mechanism may refer with high levels of HCG, which can induce hyperthyroidism [6]. In contrast, we firstly found thyroid hormones decreasing and NTIS prevalence increasing during chemotherapy in breast cancer patients, however, growth of breast cancer cells is regulated by thyroid hormones [34], thyroid hormones are considered to be the growth factor for glioma and thyroid cancer [35], the absence of thyroid hormones in cells could provoke a proliferation arrest in G0-G1 [36] or weak mitochondrial activity [12], which makes tumor cells insensitive to chemotherapy.

Basing on these points, we performed T_3_ chemosensitization test in breast cancer cells. The normal FT_3_ concentration in human body is 2.2-4.2 ng/ml, we take the physiological concentration range of FT_3_ as the reference standard. Therefore, 4 ng/ml T_3_ was used firstly in cell culture medium which contains no thyroid hormones, to evaluate whether the physiological concentration of T_3_ can elevate the chemotherapeutic efficacy. 5-Fu, the chemotherapeutic regent mainly focusing on tumor cells in S phase, is a usual component in chemotherapy for breast cancer. The T_3_ pretreatment induced MCF-7 increased progression from G0-G1 phase to S phase, with elevated chemosensitivity to 5-Fu, the similar result was found in MDA-MB-231. Taxol works differently by arresting the breast cancer cells in G2-M phase, it enhances the polymerization of tubulin to stable microtubules and also interacts directly with microtubules, stabilizing them against depolymerization by cold and calcium, which readily depolymerize normal microtubules, so cells treated with taxol are unable to form a normal mitotic apparatus. To prove T_3_ can sensitize the breast cancer cells to Taxol, we used 24 ng/ml T_3_ to pretreat MCF-7 or MDA-MB-231, the results showed that both MCF-7 and MDA-MB-231 pretreated with T_3_ were more sensitive to Taxol than those with Taxol only, without T_3_ pretreatment.

Both MCF-7 and MDA-MB-231 express the thyroid hormone receptor, the chemosensitization role of T_3_ in these two cell lines is thought to be mediated by the proliferation efficacy of T_3_. In MCF-7, an estrogen receptor positive breast cancer cell line, the proliferative effect of thyroid hormone is not only initiated through the combination with the thyroid hormone receptor, but also through the combination with the estrogen receptor [37]. There are specific mechanisms that explain why thyroid hormone stimulate cell proliferation in gastric cancer cells, the cyclin/cyclin-dependent kinase levels and activity are involved [11]. Dinda et al. [38] found thyroid hormone not only enhances the phosphorylation of pRb, but also induces the c-Myc expression and mutant P53 expression, this can also explain the cell proliferation efficacy of thyroid hormone. MDA-MB-231 is acknowledged harboring P53 mutations, which further demonstrates that thyroid hormone may promote the cell proliferation. Furthermore, thyroid hormone was found to sensitize the mitochondrial pathway to enhance the chemotherapeutic efficacy in MDA-MB-231 [12].

Thyroid hormone exerts its non-genomic and genomic actions in breast cancer cells [12]. For non-genomic action, which is characterized by combination of thyroid hormone and membrane receptors such as ER to elucidate the downstream signaling cascade, and is independent of transcriptional activity [39]. For genomic action, thyroid hormone primary mode is by binding to the thyroid hormone receptor in the nucleus then influencing the transcription and expression patterns of target genes. In MCF-7, which expresses ER and thyroid receptor, the chemosensitization role of T_3_ is mediated through the genomic and non-genomic actions of T_3_, while in MDA-MB-231, which expresses thyroid receptor, the enhanced chemotherapeutic efficacy can also be contributed to the genomic and non-genomic actions of T_3_, like the influence on genes expression and the elevated mitochondrial activity mentioned above.

In light of the findings mentioned above, T_3_ may be used as adjuvant therapy for breast cancer patients in the future, the main points for its application are usage dose and how to combine it with chemotherapy. To imitate different usage modes of T_3_ in clinic, six individual groups with treatment by T_3_ and 5-Fu were set up to separately compare with control group treated with 5-Fu only. It is indicated in this results that, to get chemosensitization efficacy, pretreatment with lower dose of T_3_, using higher dose of T_3_ together with 5-Fu or during chemotherapy with 5-Fu were all thought to be available. In clinic, however, using higher dose of T_3_ will increase the risk of suffering thyroid crisis, and in most of T_3_ chemosensitization tests from 4 ng/ml to 32 ng/ml, the relative inhibition ratio in 1st group was highest, except T_3_ at 16 ng/ml and 24 ng/ml. So, pretreatment with lower dose of T_3_ until the end of chemotherapy may be a safer and more efficient therapy for breast cancer patients. In addition, only a suitable dose of T_3_ could have chemosensitization role in chemotherapy, treatment with extremely low dose of T_3_ for long time (72 h) still could not enhance the chemosensitivity of MCF-7 to 5-Fu.

Using T_3_ for chemosensitization in breast cancer therapy is to simulate the high thyroid function in choriocarcinoma patients during chemotherapy, based on the new findings that thyroid hormones decreasing during chemotherapy, in which chemotherapeutic regents mainly take effect.

In summary, we studied the thyroid hormones and NTIS in breast patients at initial diagnosis and during chemotherapy, in which with a thyroid function significant decreasing, and found T_3_ enhanced the chemosensitivity of MCF-7 to 5-Fu. Moreover, we think the chemotherapeutic efficacy partly depends on the thyroid function, checking the thyroid function in breast cancer patients is important for elevating chemotherapeutic efficacy.

## Conclusion

**Figure 4:**
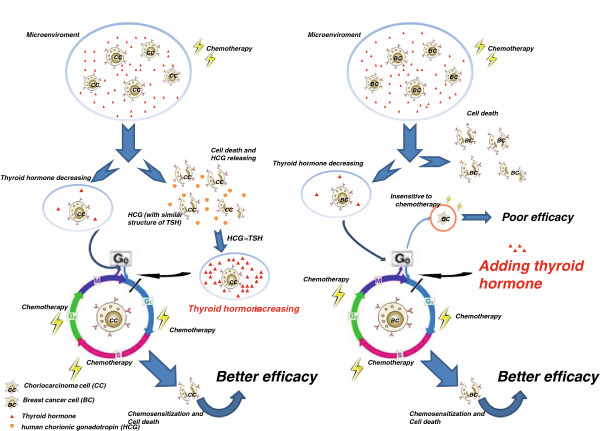
**Possible mechanism of choriocarcinoma chemotherapy and chemosensitization role of thyroid hormone in breast cancer.** Choriocarcinoma has better chemotherapeutic efficacy, the possible cause may be related with high HCG releasing when most choriocarcinoma cells (CC) are killed by chemotherapy, besides those stay in low metabolism level, which permits them from cytotoxicity effect on some level. As HCG has TSH like effect, thyroid hormone increases and promotes CC retardant in G0 phase stepping into cell division cycle, which makes CC more sensitive to chemotherapy. However, in breast cancer, thyroid hormone decreasing occurs in most breast cancer patients when chemotherapy is applied, but there is no spontaneous complement of thyroid hormone in microenviroment of breast cancer cells (BC). The absence or lack of thyroid hormone may induce G0 arrest, which makes residual BC insensitive to subsequent chemotherapy. So, inexistence of thyroid hormone decreasing in choriocarcinoma, even thyroid hormone increasing during chemotherapy, inspires us to imitate this thyroid hormone changing model in breast cancer. As we expect, the chemosensitivity can be enhanced by adding thyroid hormone before and / or during chemotherapy, which is elaborately illustrated in our cell based experiment.

Therefore, it was hypothesized, in this article, that through imitating the high thyroid function or high endocrine hormone state of some choriocarcinoma patients during chemotherapy, the chemosensitivity may be enhanced by giving thyroid hormones or other endocrine hormones to the patient before and / or during chemotherapy, which may bring a new therapy to breast cancer and other malignant tumors. This new therapy, extremely different from the traditional concept of endocrinochemotherapy (i.e., chemotherapy plus endocrinotherapy), might be termed as hormono-sensitizing chemotherapy (HSCT), endocrinosensitizing chemotherapy (ESCT), or neo-endocrinochemotherapy (NECT). The detailed mechanisms are included in Figure 4[40]. If the hypothesis mentioned above proves to be clinically safe and feasible, NECT might replace the current chemotherapy in future.

## Abbreviations

NTIS: Non-thyroidal illness syndrome; T3: Triiodothyronine; T4: Thyroxine; rT3: Reverse triiodothyronine; FT3: Free triiodothyronine; FT4: Free thyroxine; TSH: Thyroid stimulating hormone; anti-TPO: Thyroperoxidase antibody; anti-Tg: Thyroglobulin antibody; 5-Fu: 5-fluorouracil; CCK8: Cell counting kit-8; ESS: Euthyroid sick syndrome; HCG: Human chorionic gonadotrophin; TRH: Thyrotropin releasing hormone; NSGCT: Non-seminomatous germ-cell tumors; HSCT: Hormono-sensitizing chemotherapy; ESCT: Endocrinosensitizing chemotherapy; NECT: Neo-endocrinochemotherapy.

## Competing interests

The authors declare that they have no competing interests. No reimbursements, fees, funding, or salary was received from an organization that may in any way gain or lose financially from the publication of this manuscript now and in the future. We don’t hold any stocks or shares in an organization that may in any way gain or lose financially from the publication of this manuscript now and in the future. We don’t hold and currently applying for any patents relating to the content of the manuscript. We didn’t receive reimbursements, fees, funding, or salary from an organization that holds or has applied for patents relating to the content of the manuscript. We don’t have any other financial competing interests. The authors declare that they have no non-financial competing interests.

## Authors’ contributions

All the authors have made substantial contribuations to this work, JH, LJ, GJ and LX equally contributed to this study. JBH performed the basic research, did statistical analysis and drafted this manuscript. LJ, GJ, LX recorded thyroid function results, CX and XX helped to collect data and analyze it. HL directly participated in the whole process throughout the research and statistical analysis. As corresponding author, LK and GR designed and coordinated this research and provided guidance throughout this process. All authors read and approved the final manuscript.

## Pre-publication history

The pre-publication history for this paper can be accessed here:

http://www.biomedcentral.com/1471-2407/13/334/prepub
